# The Role of Radiotherapy in Octogenarian Cancer Patients

**DOI:** 10.3390/cancers17233758

**Published:** 2025-11-25

**Authors:** Aneta Lebiedzińska, Ewa Wasilewska-Teśluk, Agnieszka Sopel, Sergiusz Nawrocki

**Affiliations:** 1 Department of Oncology, Collegium Medicum, University of Warmia and Mazury in Olsztyn, Aleja Wojska Polskiego 37, 10-228 Olsztyn, Poland; 2 Department of Radiotherapy, Clinical Hospital of the Ministry of Internal Affairs and Administration with the Warmia-Mazury Oncology Centre, 10-228 Olsztyn, Poland

**Keywords:** radiotherapy, octogenarians, older adults, overall survival, toxicity, cancer

## Abstract

The use of radiotherapy in oncology patients aged 80 years and older represents a significant clinical challenge due to the limited availability of prospective data and the under-representation of this age group in clinical trials. Most existing evidence originates from retrospective analyses of small patient cohorts. Radiotherapy remains a cornerstone of cancer treatment, providing both curative and palliative benefits. Technological advances, including intensity-modulated radiotherapy (IMRT) and stereotactic body radiotherapy (SBRT), have significantly improved treatment precision, reduced toxicity, and enabled the adoption of hypofractionated regimens. These shortened treatment schedules are particularly advantageous for older patients, reducing the burden of prolonged therapy while maintaining therapeutic efficacy. Comprehensive Geriatric Assessment (CGA) plays a crucial role in optimizing treatment decisions for octogenarian patients by addressing their clinical, functional, and psychosocial needs. However, the routine use of CGA remains limited due to logistical and time constraints. Despite these challenges, current evidence suggests that radiotherapy is generally well tolerated in this population, with acceptable toxicity profiles even among frail patients. As the global population of individuals aged 80 years and older continues to increase, the development of specific clinical guidelines tailored to this demographic has become essential. There is an urgent need for prospective studies providing robust evidence regarding the efficacy, safety, and tolerability of radiotherapy in older adults. This review aims to summarize the current state of knowledge on the role of radiotherapy in patients aged 80 years and older and to highlight the need for evidence-based, individualized oncologic care for this growing patient population.

## Simple Summary

The number of cancer patients aged 80 years and older is steadily increasing worldwide, creating new challenges for oncology. This review is based on recent literature and summarizes current knowledge about the role of radiotherapy in elderly patients. Older individuals are often under-represented in clinical trials, resulting in limited data to guide optimal treatment decisions. Nevertheless, radiotherapy remains one of the most effective and well-tolerated cancer treatments, both for curative and palliative purposes. Technological progress, including modern radiotherapy techniques, has improved treatment precision and reduced side effects, allowing the use of shorter and more convenient treatment schedules. This paper emphasizes the importance of individualized treatment planning and comprehensive geriatric assessment to ensure both safety and efficacy. It also highlights the need for further studies and the development of clinical guidelines to optimize radiotherapy for this growing group of elderly cancer patients.

## 1. Introduction

The population of people over 80, called octogenarians, increases in size in the demographic structure of numerous societies worldwide [1].

According to the projections of the Statistics Poland, the number of people in Poland aged 80 and older will systematically increase. In 2020, the population of people in this age group was about 1.7 million, which constituted 4.4% of the total population. By 2050, this number is expected to increase to about 3.5 million, which will constitute 10.4% of the entire population. This means that in 2050, every tenth inhabitant of Poland will be 80 or older. The latest Statistics Poland data on life expectancy in 2023 in Poland showed that the average life expectancy was increasing. It was 74.7 years in men and 82 in women [2].

According to the International Agency for Research on Cancer, globally, the number of new cancer cases could increase by 77% by 2050, reaching 35 million annually [3].

Most tumors are diagnosed in people over 65 years of age [4].

The projections indicate a significantly aging population, an increase in the incidence of neoplastic disease, which will significantly impact the health care system and other socio-economic aspects.

The specificity of oncological treatment in the group of geriatric patients requires special attention. Comorbidities, reduced physiological reserve, general condition and a potentially higher risk of toxicity of various oncological treatment methods currently pose a great challenge related to the selection of an optimal therapy in such patients.

Radiotherapy, along with surgery and systemic treatment, is one of the primary treatment modalities for tumors. This method uses ionizing radiation to destroy tumor cells. The biological effect of ionizing radiation is DNA damage that may lead to cell death. Such effects are associated with cellular radiosensitivity and occur both in tumor cells and in normal tissue cells. Tumor cells, due to their high ability to divide and impaired repair mechanisms, are more sensitive to radiation than healthy cells. Radiotherapy is one of the key elements of oncological therapy, used both in radical and palliative treatment. As technology advances and radiotherapy becomes more precise, this treatment modality is safe, even for patients at advanced age.

Being a non-invasive local treatment method and with great potential for therapy individualization, radiotherapy may be a particularly attractive treatment option for patients. However, the question of the effectiveness, tolerability and safety of irradiation treatment in older adults remains important as they are often excluded or constitute a low percentage of patients participating in randomized clinical trials.

The aim of the study was to provide an up-to-date overview of the use of radiotherapy in geriatric patients with neoplastic diseases, with particular emphasis on patients over 80 years of age.

## 2. Materials and Methods

**Figure 1:**
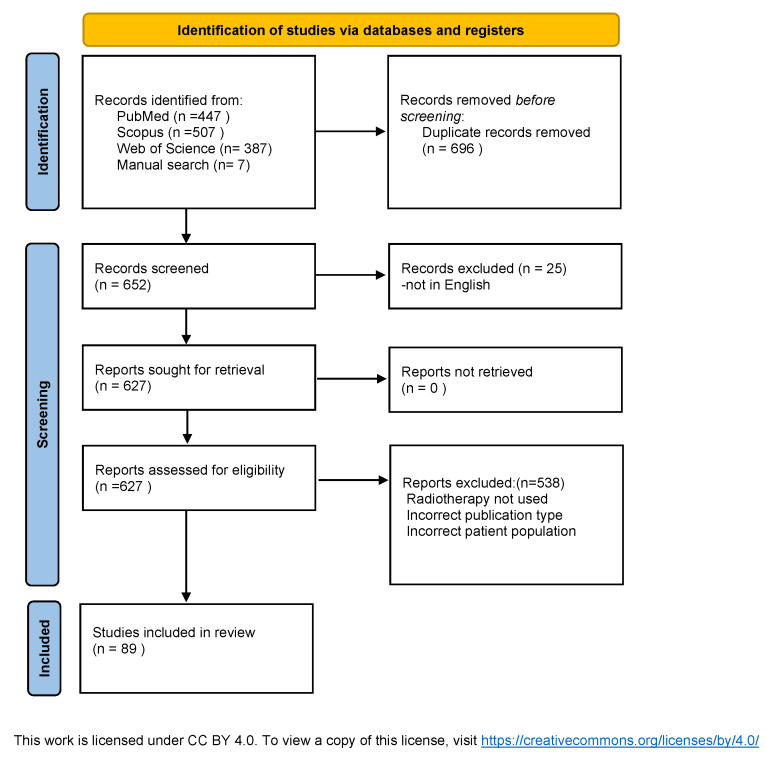
PRISMA 2020 search strategy.

The review article was prepared based on an analysis of publications retrieved from available scientific databases, including PubMed, Scopus, and Web of Science, with particular emphasis on studies published over the past 20 years. The literature review included clinical and experimental studies addressing the role, efficacy, and tolerability of radiotherapy in older adults. Only peer-reviewed articles written in English and relevant to the topic were included. The collected data were critically analyzed and synthesized to present the current state of knowledge, highlight technological advances, and identify gaps requiring further research. The literature review was conducted in accordance with the PRISMA 2020 guidelines [5], as illustrated in Figure 1. The following keywords were used during the record search: geriatric radiotherapy, elderly patients, cancer. Inclusion criteria was original research articles on radiotherapy and oncology.

Exclusion criteria was case reports, letters to the editor, commentaries. This study focuses on cancers of the breast, lung, prostate, colorectal cancer, head and neck cancers, and glioblastoma with particular consideration of metastases to the central nervous system.

## 3. Discussion

### 3.1. Breast Cancer

Patients who have breast cancer after 80 years of age usually experience a less aggressive course of the disease, but they are clinically diagnosed at more advanced stages of the disease than younger women, which is related to the lack of screening in this age group. Histologically, luminal cancers are more common [6,7]. Their incidence increases with age, while the percentage of basal-like breast cancer in older women decreases [8]. Over 80% of breast cancer cases occurring in women between 80 and 84 express the estrogen receptor (ER), compared to 60% in younger patients [9]. The luminal A subtype is often associated with a better prognosis [10]. It is characterized by slower progression and an indolent course. The presence of hormone receptors, i.e., the ER and PR (progesterone receptor) is not only an indicator of a better prognosis, but it also indicates a good response to hormone therapy [11]. Bastiaannet et al. studied a group of >120,000 women and showed that the percentage of surgery in early breast cancer in the group below 80 years of age was >93% and in the group of octogenarians, the average was 74%. The following age groups receive RT: 75–79 (86.3%), 80–84 (70.8%), 85–89 (35.9%), 90+ (14.6%) [12]. In 2020, Bertolo et al. published the results of an analysis of 97 radically treated women aged 80 and older. The control group consisted of patients aged 65–75 years. They observed that the use of radiotherapy was reduced in the group over 80. In the octogenarian group, 51.5% of patients underwent breast-conserving surgery (BCS) and 33% underwent radiotherapy [7]. In 2024, Fonseca et al. published a meta-analysis including almost 40,000 patients over 70 with breast cancer. The median age was 77.6 years. In the whole group of patients, 44.77% had clinical stage II of breast cancer (CS II) and 29.65% had CS III. BCS was performed in 32.92% and radiotherapy was used in 40.3% of the patients. The authors analyzed 17 studies published in the years 2013–2023. The analysis yielded only 3 studies in which the median age was over 80 years with the total of only 172 women. This shows how little knowledge is currently available regarding this subpopulation. The overwhelming majority of the patients had CS II or CS III cancers, and radiotherapy was implemented in 3 to 25% of the groups. No data on the use of geriatric assessment were available in any of the studies included in the meta-analysis [13].

It is currently accepted that in radical treatment, the standard of management should not deviate from generally accepted principles of breast cancer treatment. According to the SIOG (International Society of Geriatric Oncology) guidelines on the use of radiotherapy for patients >70 years of age as of 2014, candidates for postoperative WBRT (whole-breast radiotherapy) after BCS for invasive cancer and high-risk DCIS (ductal carcinoma in situ) should be in a good general condition (fit). Patients aged 50 and older who do not require lymph node irradiation, are candidates for shortened treatment regimens. Post-mastectomy irradiation should be considered in older patients with pT3-4 tumors or with ≥4 tumor-positive axillary nodes [14]. Postoperative whole-breast radiotherapy is the standard of care for all able-bodied patients following breast-conserving surgery. A combination of BCS and WBRT was associated with a two-fold reduction in recurrence even in older patients, although the absolute 10-year reduction in the risk of locoregional or distant recurrence was found to be lower in women ≥70 years of age and was 8.8% compared to younger women (17.7%) [15]. In older patients, shorter treatment courses using higher fractional doses [hypofractionation (HF)] constitute an alternative to WBRT. Studies evaluating different HF-RT regimens after BCS revealed that HF regimens offered similar local control rates, and comparable toxicity [16,17]. Due to the reduced treatment time, shortened irradiation regimens are an attractive form of therapy, especially for older patients, for whom the distance between the radiotherapy center and their place of residence often constitutes an obstacle in the implementation of treatment. Currently, shortened dose fractionation regimens are recommended both for older and younger patients, which stays in line with the results of the FAST-Forward study. However, patients older than 80 years constituted only 3% of the group [18].

When planning radiotherapy, special emphasis should be placed on the need for modern 3D and 4D planning methods with the use of cardiac protective treatment techniques, deep inspiration breath hold technique, which facilitates limiting the doses in the heart and is an important factor in the group of older patients at a higher risk of ischemic heart disease. In case of post-mastectomy radiotherapy, data are insufficient to suggest that survival gains might include women >70 years of age [19]. A 2014 meta-analysis by the Early Breast Cancer Trialists’ Collaborative Group (EBCTCG) revealed that post-mastectomy radiotherapy reduced 20-year breast cancer mortality by 7.9% in patients with three or fewer positive nodes and by 9.3% in patients with four or more positive nodes [20]. The role of RT in patients with three or fewer positive lymph nodes remains a topic of debate [21].

Accelerated partial breast irradiation (APBI) combines the administration of a higher fractional dose, short treatment duration, and a small target volume limited to the tumor bed. IORT (intraoperative radiotherapy with electrons) is an attractive option in older women because it may be administered as a single dose. Two randomized trials on APBI were published. The TARGIT-A study included 3451 patients with the minimum follow-up of 4 years for 1010 patients and 5 years for 611 patients. The absolute difference in local recurrence at 5 years was statistically significant and was 2% higher in the IORT group (3.3%) compared to the WBRT group (1.3%). Grade 3 toxicity was lower in IORT subjects (0.5% vs. 2.1%). Patients older than 74 accounted for about 8% of the participant [21,22,23]. The ELIOT study included 1305 patients up to 75 years of age with T1–T2 breast cancer (max. up to 2.5 cm) pretreated with BCS. They were randomly assigned to groups receiving postoperative whole-breast irradiation or a single dose (21 Gy) of IORT. The local recurrence rate was 4.4% in the IORT group and 0.4% in the WBRT group with the median follow-up of 5.8 years [24]. The EORTC study compared WBRT with or without an additional radiation dose per tumor bed. The reduction in the risk of local recurrence after the addition of radiation boost was not statistically significant in patients aged 60 years and older. Therefore, boost was recommended in this age group only for patients at a higher risk of relapse [25]. In patients older than 70 with a tumor <2 cm, positive hormone receptors, after BCS, supplementary radiotherapy may be discontinued if a minimum of 5 years of hormone therapy is planned [26]. A meta-analysis conducted by the EBCTCG showed that post-BCS RT reduced the recurrence rate, but the absolute benefit in the older, lower-risk group receiving endocrine therapy was limited [15,20]. The CALGB 9343 study showed that, in women over 70 years of age, the local recurrence rate after 12 years was 10% without radiotherapy compared to 2% with radiotherapy. However, it did not affect the overall survival (OS) [27].

According to current guidelines of the National Comprehensive Cancer Network (NCCN, version 6.2024), it is recommended to consider the omission of radiotherapy after BCS if adjuvant endocrine therapy is planned and patients meet the following criteria: age ≥ 70 years, HR+, HER2-negative, cN0, pT1 and age ≥65 years, HR+, HER2-negative, pN0, tumor ≤ 3 cm in diameter [28].

The preliminary results of a phase III EUROPE study were published in 2025. The study was conducted in a group of patients >70 years of age undergoing BCS for early luminal A breast cancer. The authors compared endocrine therapy and RT in the context of the quality of life after 2 years of follow-up. Patients after RT reported a better quality of life compared to patients undergoing endocrine therapy. Adverse reactions were reported more frequently after endocrine therapy (85% vs. 67%). Endocrine therapy was discontinued in 12% of the patients due to adverse reactions. The study presented preliminary data. The authors suggested that RT might constitute an alternative to several years of endocrine therapy in a certain group of patients. Patients over 80 years of age accounted for 26–28% of the study group [29].

In 2021, joint recommendations were published by two scientific societies, the European Society of Breast Cancer Specialists (EUSOMA) and the SIOG for patients >70 years of age. Whole-breast radiotherapy after breast-conserving surgery remains the standard of care for most older patients and reduces the risk of disease recurrence by half. The benefit of radiotherapy in the group of elderly patients with positive hormone receptors and low-grade cancer is moderate.

WBRT remains the standard of care for older post-BCS patients. The omission of radiotherapy in low-risk patients may be safe and justified (level 1 recommendation). In patients over 60 years of age, it is recommended to use an additional dose of radiation (boost) only in the group at a higher risk of relapse (level 1).

Hypofractionated regimens are recommended (40 Gy in 15 fractions over 3 weeks, or 42.5 Gy in 16 fractions over 3.5 weeks, or 26 Gy in 5 fractions over 1 week) (level 4). Partial breast irradiation (PBI) is recommended in women ≥50 years of age with grade 1–2 tumors, pN0, HER2-negative, hormone receptors positive, ≤30 mm in diameter, and radial margins ≥1 mm (level 4). The role of post-mastectomy RT in patients with one to three positive lymph nodes remains controversial [30].

The use of radiotherapy in the old patient with breast cancer are presented in Table 1.

**Table 1:** Summary of radiotherapy in breast cancer patients.

References	Radiotherapy Approach	Outcomes/Tolerability	Guidelines
Fonseca et al. [13]	Meta-analysis of RT in >70 yrs	In >80 yrs RT used in only 3–25%; no geriatric assessment	Need for improved evaluation of older adults
Murray Brunt et al. [18]	Post-BCS RT; hypofractionation	Comparable control and toxicity; ≥80 yrs = 3% of cohort	Recommended HF-RT 26 Gy/5 fractions
McGale et al. [20]	Post-mastectomy RT (PMRT)	Limited data in >70; 20-year mortality reduction 9.3% with ≥4 nodes	Standard for ≥4 positive nodes
Vaidya et al. [22,23] Veronesi et al. [24]	Partial RT (APBI, IORT) vs. WBRT	Higher recurrence with IORT (3.3% vs. 1.3%), lower toxicity	Boost recommended only for high-risk ≥60 yrs
Huges et al. [26,27]	RT omission after BCS in >70 yrs	12-year recurrence 10% without RT vs. 2% with RT; no OS difference	RT may be omitted in ER+ with endocrine therapy
Rashmi et al. [28]	Criteria for RT omission after BCS	RT omission possible: ≥70 yrs, HR+, HER2–, pT1, cN0	NCCN v6.2024
Meattini et al. [29]	RT vs. endocrine therapy	Better QoL after RT; ≥80 yrs = 26–28%	RT as alternative to long-term endocrine therapy
Biganzoli et al. [30]	RT after BCS; HF and PBI	RT halves recurrence risk; may be omitted in low-risk	EUSOMA–SIOG 2021

### 3.2. Lung Cancer

Lung cancer is the leading cause of cancer death in the world [31]. Surgical treatment remains the gold standard in the treatment of NSCLC stages I and II. Huang et al. [32] conducted a study in the Chinese population including a group of 7861 patients older than 80. The authors showed that lobectomy could be used in patients aged 80–85 years if the general condition of the patient allowed. Li et al. carried out a study in a group of patients >80 with the tumor size up to 4 cm and pleural infiltration. They reported that the results of wedge resection were comparable to those following anatomical resection. Multivariate analysis showed no significant differences in cancer-specific survival (CSS) or overall survival between both surgical groups [33]. Radiotherapy is widely used in patients with lung cancer in both non-small cell lung cancer (NSCLC) and small cell lung cancer (SCLC). It may be successfully implemented in patients over 80 years of age. The study conducted in a group of octogenarians with stage IA non-small cell lung cancer revealed that surgery with lymph node dissection provided better OS outcomes than radiotherapy. The 1-, 3- and 5-year OS rates were 90.0%, 76.9%, 59.9% for surgery and 86.0%, 54.3%, 28.0% in the radiotherapy group, respectively. CSS survival was superior in the surgical group compared with the radiotherapy group. However, in the discussion section, the authors emphasized a possible impact of the negative selection in case of the group qualified for RT. Factors such as perioperative mortality, general condition or comorbidities were not analyzed, and they could have a significant impact on the selection of treatment modality and the results obtained [34]. Robinson et al. compared lobe resection (lobectomy or pneumonectomy) with stereotactic radiotherapy (SBRT) in stage I NSCLC patients. The overall survival (OS, 63.5% vs. 29.6%) was greater for lobe resection, although specific survival rates were comparable (CSS, 81.3% vs. 75.3%). Elderly patients, at the median age of 76 years and more internally burdened patients were eligible for SBRT, while in case of surgery, the median age was 66. Total postoperative mortality was 1.9%, including the 30-day mortality of 1.5% [35]. High postoperative mortality was also found in the study by Stokes et al. in patients >80 years of age: 30 days: 3.03%; 90 days: 3.67%. Differences in the 30- and 90-day post-treatment mortality between surgery and SBRT increased depending on age, with the largest differences in favor of SBRT being observed in patients over 70 years of age [36]. An attempt to conduct a randomized SABRTooth trial in patients with stage I NSCLC comparing surgery in patients at an increased risk of complications and SBRT was discontinued due to the lack of recruitment. The median age was >75 years in the group. A significant proportion of patients randomly assigned to the surgical group refused the procedure and chose SBRT [37].

Stereotactic radiotherapy is an effective treatment modality in patients over 80 with early-stage non-small cell lung cancer. The most benefits were noted in patients with KPS (Karnofsky Performance status) ≥70 [38]. Those observations were confirmed by a retrospective analysis of patients aged ≥80 diagnosed with early-stage non-small cell lung cancer (T1-T2b) treated with SBRT. The study evaluated treatment effectiveness by analyzing indicators such as local recurrence-free survival (LRFS), regional recurrence-free survival (RRFS), progression-free survival (PFS), and overall survival (OS). The average age in the group was 85 years. The three-year survival rates were: OS—65.3%, PFS—58%, RRFS—73.9% and LRFS—85.3%. Multivariate analysis showed that tumor size, general condition, histological type, and pre-treatment C-reactive protein (CRP) value had a significant impact on OS results [39].

Several retrospective subgroup analyses of randomized trials evaluated the effects of concurrent radiochemotherapy (CRT) in elderly patients compared to younger patients, yielding inconsistent results. Subgroup analyses of several phase I-III studies conducted by the Radiation Therapy Oncology Group (RTOG) on radiotherapy alone, sequential CRT, and concurrent CRT showed that concurrent CRT did not improve outcomes and was associated with greater toxicity in patients >70 years of age [40,41]. A total of 12,641 patients with CS III were analyzed. Radiotherapy alone compared to CRT was associated with poorer OS. Age >70 years was a negative prognostic factor. The obtained results may have been influenced by the lack of comprehensive geriatric assessment prior to qualification for treatment.

In elderly patients with advanced CS III disease, radiotherapy as monotherapy is often considered as a radical treatment. Low toxicity and long-lasting local effects, including good symptom control, mean that it may be successfully used in older patients. Pergolizzi et al. conducted a study in a group of 40 patients with CS IIIA and the median age of 77 years (75–83). They used RT as monotherapy at a median dose of 60 Gy (range 54–64 Gy), 2.0 Gy per fraction. In the study, 3-year OS was achieved at the level of 18%. Mild esophagitis was observed in 70%. Two patients experienced clinical radiation pneumonia [42]. Geinitz et al. observed reduced esophageal tolerability in older patients treated with chest irradiation [43]. Lonardi et al. evaluated the total dose of 50 Gy (1.8–2.0 Gy per fraction) in a similar group of patients with CS IIIA/B aged 75–85. A 2-year OS was reached at the level of 10%. However, it should be emphasized that the applied total dose of 50 Gy was suboptimal [44].

Carbon ion radiotherapy (CIRT) is effective and safe for octogenarians with locally advanced non-small cell lung cancer. It is an advanced form of hadron therapy using high-energy carbon ions, offering higher biological effectiveness than photons and protons and therefore providing an effective treatment option for radioresistant and hypoxic tumors. However, the availability of this method is very low. Hayashi et al. examined 32 patients over 80 years of age who were treated with carbon ion radiotherapy only. Patients in CS IIA, IIB, IIIA and IIIB were included in the study. The CIRT dose was 72.0 Gy and the median follow-up was 33.1 months. All patients completed CIRT therapy. No Grade ≥4 toxicity was observed. The rate of 2-year local control (LC) was 83.5%, progression-free survival was 46.7% and the overall survival was 68.0% [45]. (Table 2).

A meta-analysis comparing proton therapy (PT) and photon therapy showed that the use of proton therapy at the early stage of NSCLC remained controversial and the evidence was insufficient to determine its superiority over photon therapy. As regards the effectiveness, no significant differences in 1-year OS, 3-year OS, and 3-year PFS were determined between PT and RT. In the case of toxicity, no significant difference was observed in treatment-related adverse events and radiation pneumonia [46].

In limited disease (LD) small cell lung cancer, the best treatment results are obtained with concurrent radiochemotherapy. However, patients >70 are rarely eligible for clinical trials with this treatment. A retrospective assessment of the therapeutic effectiveness and safety of CRT in elderly patients (≥75 years of age) with LD small cell lung cancer was published in 2024 by Ayako Shiono et al. They showed that no significant differences in PFS or OS occurred between the concurrent and sequential CRT groups. Concurrent treatment is more difficult to perform and more toxic. Sequential CRT may be considered as the treatment of choice for such patients [47]. The CONVERT study established hyperfractionated irradiation at 45 Gy/21 days with platinum-based chemotherapy as the standard of care. Patients over 80 years of age experienced greater toxicity (Grade ≥3). The number of patients was low, which makes it difficult to draw final conclusions. Considering that probably the healthiest group of octogenarians was chosen, the need for caution in offering CRT in this age group was suggested [48].

Prophylactic cranial irradiation (PCI) was found not to improve the 2-year OS and should not be used routinely in a group of patients >70 and lung tumor size >5 cm [49].

**Table 2:** Summary of radiotherapy in lung cancer patients.

Reference	Radiotherapy Approach	Outcomes/Tolerability	Guidelines/Authors’ Comments
Bei et al. [39]	SBRT (T1–T2b) in ≥80 yrs	3-yr: OS 65.3%, PFS 58%, RRFS 73.9%, LRFS 85.3%	SBRT effective; incorporate geriatric assessment and biomarkers
Pergolizzi et al. [42]	RT monotherapy (median 60 Gy, 2.0 Gy/fx)—stage IIIA, median age 77	3-yr OS 18%; mild esophagitis 70%;	Definitive RT monotherapy is a radical option for selected patients
Lonardi et al. [44]	RT 50 Gy (1.8–2.0 Gy/fx)—stage IIIA/B (75–85 yrs)	2-yr OS 10%; total dose considered suboptimal	Consider higher/definitive doses in fit patients
Hayashi et al. [45]	CIRT (carbon ions) for locally advanced NSCLC in >80 yrs	All completed therapy; no ≥G4 toxicity; 2-yr LC 83.5%, PFS 46.7%, OS 68%	CIRT safe and effective but with very limited availability
He et al. [46]	Proton therapy vs. photon RT in early-stage NSCLC	No significant differences in 1- and 3-yr OS or 3-yr PFS; similar toxicity	Evidence insufficient to claim superiority of proton therapy in early-stage disease
Shiono et al. [47]	LD-SCLC: concurrent vs. sequential CRT in ≥75 yrs	No PFS/OS differences; concurrent more toxic	Offer concurrent CRT in fit octogenarians
Christodoulou et al. [48]	LD-SCLC: standard hyperfractionation 45 Gy/21 days + platinum chemo	In >80 yrs higher ≥G3 toxicity; small sample	PCI should not be routine in this subgroup
Kim et al. [49]	PCI in >70 yrs with tumor >5 cm	No 2-yr OS improvement; not recommended routinely	

### 3.3. Prostate Cancer

In 2040, the estimated incidence of prostate cancer (PC) in the world will be 2.43 million new cases. The number of patients over the age of 80 will double and reach approximately 0.5 million new cases [1]. Patients whose life expectancy is >10 years should be eligible for radical prostatectomy, so the treatment modality is not a preferred one in the octogenarian group. Radiotherapy occupies an important role in this age group. (Table 3).

In low-risk patients with the expected survival of <10 years, the “watch and wait” or active surveillance strategy should be considered [50]. Patients in higher-risk groups with the life expectancy <10 years should receive ADT (Androgen Deprivation Therapy) to alleviate disease symptoms [50]. Geriatric assessment facilitates appropriate qualification for the treatment of older adults with prostate cancer. A better understanding of the role of active surveillance for less aggressive disease also contributes to the individualization of care [51]. The evaluation of the benefits of the applied management strategy, active or conservative treatment and the probability of death due to any cause within 10 and 15 years may be estimated using the PREDICT Prostate tool. An 80-year-old patient with comorbidities has about a 15% chance of surviving 10 years regardless of cancer. The respective percentage in an 85-year-old patient is about 4% [52]. Comorbidities in patients >75 should be a relative contraindication to aggressive treatment in low-risk PC [53]. Nguyen et al. analyzed the oncological results of a small, multicenter cohort of 65 elderly patients at risk (>80 years) with comorbidities requiring treatment, treated with 3D radiation (45 Gy to the pelvis; and 69.5 Gy to the prostate). The authors observed the 5-year OS rate of 77% [54]. Okonogi et al. compared the use of IMRT (Intensity-Modulated Radiation Therapy) at a dose of 78 Gy (+ADT) for the median duration of 17 months in 23 patients >80 years compared to 171 younger patients in the intermediate- or high-risk group. The 3-year OS of 92% was achieved compared to 99.4% in the group <80. However, the small size of the groups did not allow binding conclusions to be drawn [55]. In 2023, Le Tuo el al. published the results of their research in a group of 107 patients >80. In the study group, 72.9% of patients had high-risk cancer, and 27.1% had intermediate-risk prostate cancer. The median follow-up was 97 months. The median dose was 78 Gy. Almost the entire group, i.e., 97.2% of patients, received androgen deprivation therapy. The 5-year OS was 91.4% and 10-year OS was 67.2%. Selected patients over 80 years of age with localized prostate cancer were confirmed to benefit from radiotherapy in combination with ADT. The rate of gastrointestinal (GI) and genitourinary (GU) toxicity was acceptable [56].

IMRT and advanced variant, VMAT (Volumetric Modulated Arc Therapy), in which beam intensity modulation occurs simultaneously with the rotation of the machine around the patient, are commonly used in the treatment of prostate cancer, as they are associated with a lower risk of Grade 3 rectal complications compared to 3D EBRT (external beam radiotherapy) and allow for a reduction in exposure time. These techniques are well suited for hypofractionation.

Hypofractionation with the 5-week 62 Gy regimen is equivalent to the conventional fractionation regimen (80 Gy for 8 weeks) in terms of acute and late toxicity. In a study including 168 individuals, the patients were randomly assigned to receive hypofractionated radiotherapy (62 Gy in 20 fractions over 5 weeks, 4 fractions per week) versus conventional fractionated radiotherapy (80 Gy in 40 fractions over 8 weeks) using the conformal technique for the prostate and seminal vesicles. No differences were observed as regards the frequency or severity of genitourinary toxicity and late gastrointestinal toxicity (GI) between both treatment regimens [57].

Hypofractionation is increasingly used in the treatment of PC, which is important in this group of patients due to the shortening of therapy duration [58]. (Table 3). In the phase III of HYPO-RT-PC study, acute toxicity was shown to be higher with ultrahypofractionation compared to conventional fractionation. The analysis of the quality of life reported by patients showed that ultrahypofractionation was tolerated as well as conventional fractionation. The dosage regimen of 78.0 Gy in 39 fractions, 5 days per week for 8 weeks was compared to ultrahypofractionation (42.7 Gy in 7 fractions, 3 days per week for 2.5 weeks). However, patients >75 were not eligible for the study [59]. A British phase III CHHIP study recruited 3216 men with stage T1b-T3a disease who were randomly assigned to receive 74 Gy in 37 fractions, 57 Gy in 19 fractions, or 60 Gy in 30 fractions (moderately hypofractionated). After 5 years of observation, it was shown that the 60 Gy regimen was not inferior to the 74 Gy regimen and was therefore recommended for use in clinical practice. The study included patients >80 years of age, but the median age of the whole group was 69 years. The dose of 60 Gy was the preferred regimen for the >69 age group. The assessment of late toxicity at 10 years confirmed no differences in three study arms [60].

Brachytherapy (BRT) is effective and safe treatment for men with early localized prostate cancer. Low Dose-Rate Brachytherapy (LDR) has high prostate cancer disease control but worse impact on urinary symptoms. High Dose-Rate Brachytherapy (HDR) provides comparable disease control with less urinary toxicity [61] The role of BRT in old adult patients with low-risk PC and the life expectancy below 10 years is controversial. Li et al. investigated acute toxicities, especially those requiring admission to the emergency department within 30 days following BRT. Age > 75 years was found to be an independent adverse risk factor for acute toxicity [62]. Age > 80 years in BRT patients was found to be an independent risk factor for late genitourinary toxicity (G ≥ 2) but the group of >80 consisted of only 35 patients out of a total of 1492 participants [63].

**Table 3:** Prostate Cancer—Radiotherapy in Old Adults.

Reference	Radiotherapy Approach	Outcomes/Tolerability	Guidelines/Authors’ Comments
Nguyen et al. [54]	3D-RT: pelvis 45 Gy + prostate 69.5 Gy in >80 with comorbidities	5-year OS 77%.	RT is feasible for selected octogenarians
Okonogi et al. [55]	IMRT 78 Gy + ADT; >80 vs. younger in intermediate/high-risk	3-year OS 92% (>80) vs. 99.4% (<80); small sample limits conclusions	IMRT + ADT feasible in old adults; larger studies needed
Le Tuo et al. [56]	RT (median 78 Gy) + ADT in >80; 72.9% high-risk; 27.1% intermediate-risk	5-year OS 91.4%, 10-year OS 67.2%; acceptable GI/GU toxicity; 97.2% received ADT	Selected >80 with localized PC benefit from RT + ADT
Arcangeli et al. [57]	Hypofractionation 62 Gy/20 fx vs. conventional 80 Gy/40 fx (3D conformal)	No differences in GU frequency/severity or late GI toxicity	HF offers shorter treatment with comparable toxicity—valuable in older adults
Fransson et al. [59]	Ultra-hypofractionation 42.7 Gy/7 fx vs. conventional 78 Gy/39 fx.	Higher acute toxicity with ultra-HF; QoL similar; >75 yrs not eligible	Use caution extrapolating to >75; evidence in the oldest is limited
Syndicus et al. [61]	74 Gy/37 fx vs. 60 Gy/30 fx vs. 57 Gy/19 fx (moderate HF)	60 Gy non-inferior to 74 Gy; no late-toxicity differences at 10 yrs	60 Gy/30 fx recommended in practice, suitable for well-selected older adults

### 3.4. Colorectal Cancer

In octogenarians, colorectal cancers occur more commonly on the right side. They secrete the CEA marker, cT3/T4 tumors are more common, but they less commonly metastasize to the lymph nodes [64].

Patients >80 are more likely to undergo life-saving surgery. They have lower survival rates and experience higher treatment toxicity [65]. A multivariate analysis of the Japanese population demonstrated that age over 80 was an unfavorable prognostic factor for the overall survival [64]. Similar observations were described for the population of Israel [66].

Chen et al. conducted a meta-analysis of 21 studies including a total of 13,790 patients with colorectal cancer qualified for surgery at the age of 80 and older. The authors observed a high incidence of general postoperative complications, a high incidence of postoperative internal complications, high in-hospital mortality and lower survival rates. Hospital mortality in patients aged 80 was 4-fold higher than in patients aged <80. Subgroup analysis revealed that bowel obstruction, respiratory failure, cardiac events, vascular and neurological complications occurred more frequently in patients aged 80 [67]. The EURECCA study showed a significant variation in the 5-year relative survival between different European countries in patients >80 with rectal cancer. The study showed differences in qualification for preoperative radiotherapy in patients with stages I–III. The percentage of preoperative radiotherapy in CS I-III patients ranged from 7.9% to 28.9% [68].

Qualification for radiochemotherapy of rectal cancer should be based on the use of geriatric scales. A study by Pasetto et al. was conducted in a group of 36 patients >70 (70–82) with CS II and III qualified for preoperative radiochemotherapy. They were assessed with the Charlston scale (from 0–2 points). The patients received 3D radiation at a total dose of 50.4 Gy (45 Gy/25 fractions over 5 weeks to the pelvis, followed by 5.4 Gy/3 fractions as tumor boost) with 5FU chemotherapy, which was discontinued in 13 patients (36%) due to toxicity. Surgery was performed in 32 individuals after 6 weeks. The median PFS was not achieved during the observation of 37 months [69]. (Table 4).

In the study by Margalit et al., 36 patients were enrolled at the median age of 79.0 years (range 75–87 years). Mild or no comorbidities were noted in 53%, and moderate or severe comorbidities were noted in 47% of the patients. Overall, 58% of patients were treated with preoperative CRT, while 33% of patients underwent postoperative CRT. Although 92% of the patients received the planned dose of radiotherapy, 25% required a break in RT treatment and 11% were hospitalized. Dose reduction, treatment interruption, or the discontinuation of chemotherapy were necessary in 33% of cases. A total of 39% of patients completed ≥4 months of adjuvant chemotherapy and 17% completed treatment as planned. The majority of older adults patients with rectal cancer required early termination of treatment, discontinuation, or cytostatic dose reduction. These data suggest that caution is necessary with further intensification of combination therapy for rectal cancer in older adults patients who require the aggressive treatment of comorbidities. Preoperative treatment was better tolerated than the postoperative one [70]. For older patients who are not eligible for chemotherapy, short radiotherapy with deferred surgery is beneficial for unresectable or borderline resectable tumors at diagnosis. In patients with a complete response after short radiotherapy, the “watch and wait” option is possible, especially important in older and frail patients [71,72]. The 5 × 5 Gy regimen with deferred surgery may be routinely used to treat older patients who are not eligible for chemotherapy for both unresectable and early-stage tumors prior to planned resection [73]. No differences in local effectiveness were observed between the 5 × 5 Gy regimen with consolidation chemotherapy and long-term chemoradiotherapy. Nevertheless, improvement in overall survival and lower acute toxicity levels were in favor of the 5 × 5 Gy regimen with consolidation chemotherapy [74]. Long-term results of the 8-year follow-up indicated the equivalence of both methods of treatment [75].

**Table 4:** Rectal Cancer—Radiotherapy & Outcomes in Older Adults.

Reference	Radiotherapy Approach	Outcomes/Tolerability	Guidelines/Authors’ Comments
Pasetto et al. [69]	Preop CRT: 3D RT 50.4 Gy (45 Gy/25 fx + 5.4 Gy/3 fx boost) + 5-FU	In >70 (70–82): chemo stopped in 36%; surgery after 6 weeks in 32/36; median PFS not reached at 37-month follow-up	Use geriatric scales (Charlson 0–2) for selection
Margalit et al. [70]	Pre- vs. postoperative CRT in old adults	92% received planned RT dose; 25% required a break, 11% hospitalized; 33% needed dose-mod/cessation of chemo; only 17% completed as planned. Preop better tolerated than postop	Avoid intensification without aggressive comorbidity management; prefer preop CRT when feasible
Socha et al. [71] Beets et al. [72]	Short-course RT 5 × 5 Gy with deferred surgery; “watch & wait” after complete response	Beneficial for unresectable/borderline- resectable and frail/chemo-ineligible; W&W feasible after CR—important in frail older adults	Consider 5 × 5 Gy + delay as organ-sparing strategy in older chemo-ineligible patients
Bujko et al. [73]	5 × 5 Gy with delay surgery also for early-stage cases prior to planned resection when chemo-ineligible	May be used routinely in this population	Standard option for older chemo-ineligible in unresectable and early-stage pre-resection settings

### 3.5. Head and Neck Cancer

The basic treatment modalities in patients with squamous cell carcinoma of the head and neck region include surgery, radiotherapy and radiochemotherapy. Qualifying patients for specific treatment options, a comprehensive assessment of overall condition is essential. Patients with head and neck cancers, regardless of age, are often cachectic and frail due to difficulties with oral intake or lifestyle-related factors.

Retrospective analysis of 260 elderly patients showed the following data concerning patients divided into age groups. Patients >80 accounted for 26% (61 individuals). The patients were qualified for surgery, RT or CRT. Patients >80 preferred RT as their primary treatment. Over half of the patients who started RT eventually discontinued the treatment. No significant difference was noted in the overall survival between the groups, regardless of the selected treatment method [76]. Older adults experienced greater acute and late toxicity as treatment was intensified. In particular, the addition of chemotherapy to radiotherapy increased toxicity and decreased therapy tolerability [77]. A meta-analysis of 19,805 patients included in 107 randomized trials concerning chemotherapy in squamous cell carcinoma of the head and neck (MACH-NC) showed that radiochemotherapy (CRT) improved the overall survival with locally advanced cancer. In contrast, age contributed to the deterioration of the treatment results in patients >70 years of age. Caution was advised in qualifying patients for combination therapy [78]. (Table 5).

Radiotherapy as monotherapy may be effective and well-tolerated by the older adults. Chronological age is not a contraindication to radical radiotherapy. Zachariah et al. retrospectively analyzed patients aged 80 years and older who received palliative or radical radiotherapy alone. A total of 203 patients aged 80–94 years were enrolled in the radiotherapy group, out of which 50 were treated for head and neck cancer. A complete response was achieved by 65% of patients treated for HNC. Response to treatment was observed in 81% of palliative patients. The toxicity profile was acceptable. Mucositis was the most common adverse reaction and occurred in 38%. Grade 3 dermatitis was reported in 2% of patients and treatment was discontinued in only 2 patients [79]. Wasil et al. analyzed 183 patients >80 who underwent radical or palliative radiotherapy. The majority of patients completed the planned treatment (77%). Treatment interruptions were required during radiotherapy. They were reported in 36% of the treated patients. Radiotherapy may be safely used in the elderly both when aiming for full recovery and during palliative treatment [80]. Desai et al. conducted a retrospective analysis of 34 patients who were ≥80 years of age at diagnosis, treated between 2012 and 2016. Demographic, clinical and treatment data were collected. Patients who received 3D-RT had the OS of 23 months, while patients undergoing single-modality RT had the OS of 8.5 months [81].

**Table 5:** Head and Neck Cancer—Radiotherapy in Older Adults.

Reference	Radiotherapy Approach	Outcomes/Tolerability	Guidelines/Authors’ Comments
Kim et al. [76]	Retrospective analysis (26% >80); comparison: surgery, RT, CRT	>80 preferred RT; >50% discontinued treatment; no OS difference between modalities	Individualized treatment selection necessary
Lacas et al. [78]	CRT vs. RT in squamous cell HNC	CRT improved OS in locally advanced disease; >70 yrs—worse outcomes	Avoid CRT in older adults; favor RT alone or less toxic regimens
Zachariah et al. [79]	Radical or palliative RT in ≥80	CR in 65% of HNC; 81% response in palliative	RT is effective and well tolerated in ≥80; age not a contraindication for radical RT
Wasil et al. [80]	Radical/palliative RT in >80	77% completed RT; 36% required interruptions; overall safe	RT feasible and effective both in radical and palliative settings in older adults
Desai et al. [81]	3D-RT vs. single-modality RT in ≥80	3D-RT: OS 23 months; single-modality RT: OS 8.5 months	Advanced techniques (3D-RT) improve OS and are well tolerated in the older adults

### 3.6. Glioblastoma

The older adults with glioblastoma multiforme (GBM) represent a clinical challenge for neurosurgeons and oncologists. However, data on treatment outcomes in patients over 80 years of age undergoing surgical treatment are limited. Abdullah et al. performed a retrospective analysis of factors that might affect the OS of patients over 80 undergoing surgical treatment. The expression of oncogenic immunohistochemical markers (EGFR, p53, IDH1) and the cell proliferation index (Ki-67) were analyzed. The median overall survival was 4.2 months in the group of 58 patients over 80 who had undergone surgery. A statistically significant correlation between overall survival and Karnofsky performance status was demonstrated. Patients who received radiochemotherapy or radiotherapy were characterized by a significantly longer survival compared to patients who did not receive any postoperative adjuvant therapy. Longer overall survival was observed in patients without EGFR or p53 expression. In the octogenarian population, a benefit in terms of the overall survival was observed in patients with higher preoperative performance status (KPS), who received postoperative adjuvant therapy and in whom p53 expression was undetected. The results may be useful in making decisions regarding the treatment of patients with glioblastoma multiforme in the group of octogenarians and also confirm the key role of EGFR and p53 expression in oncogenesis, especially in the context of advanced age [82].

In 2024, Malmström et al. published a meta-analysis of data obtained from three clinical trials: Nordic, NOA-08 and CE.6. The meta-analysis included a total of 1277 people at the median age of >70 (range 60–90 years) [83]. Nordic and NOA-08 studies compared temozolomide (TMZ) therapy with RT in patients over 60 and 65 years of age. The Nordic study showed the superiority of TMZ compared to standard RT. Moreover, hypofractionated RT was shown to improve the overall survival compared to standard RT in patients >70 [84]. The NOA-08 study compared TMZ and RT and showed TMZ equivalence compared to standard RT [85].

In the CE.6 study, patients with glioma aged >65 had better OS in case of hypofractionated RT (40 Gy/15 fractions) with adjuvant TMZ compared to RT as monotherapy [86] (Table 6). The meta-analysis showed that the coexistence of hypertension, diabetes and/or stroke deteriorated survival rates in patients treated with TMZ. The prognostic factors influencing the OS included: the scope of resection, general condition according to the WHO, age, and the use of glucocorticosteroids. It was confirmed that the methylation state of the MGMT promoter was a predictive factor of response to TMZ [83].

Particular attention as regards the subpopulation of the elderly was attributed to the use of shortened irradiation treatment regimens. (Table 6). A study included 100 GBM individuals aged >60 who were postoperatively randomly assigned to receive standard radiotherapy (60 Gy in 30 fractions over 6 weeks) or a shorter course of radiotherapy (40 Gy in 15 fractions over 3 weeks). The overall survival was the primary endpoint of the study. The overall survival was comparable and equaled 5.1 months for standard RT versus 5.6 months for 40 Gy. The probability of surviving 6 months was also similar and equaled 44.7% for standard RT compared to 41.7% in the hypofractionated arm. As regards patients who completed RT as planned, 49% of patients (standard RT) compared to 23% required an increased post-treatment corticosteroid dose. The study confirmed the effectiveness of the regimen involving hypofractionation [87]. Another study in a group of elderly patients, in a poorer general condition, with newly diagnosed glioma, compared short-term radiotherapy of 25 Gy in 5 daily fractions for 1 week to the 40 Gy radiotherapy regimen in 15 fractions for 3 weeks. Short-term radiotherapy was not inferior to commonly used radiotherapy. The median PFS was 4.2 months in both arms. The median overall survival was 7.9 months in the 25 Gy arm and 6.4 months in the 40 Gy arm. The quality of life was comparable in both groups. A short radiotherapy regimen may be recommended as a treatment option for old adults and/or frail patients with newly diagnosed glioblastoma multiforme [88].

Single-agent TMZ chemotherapy may constitute an alternative to RT. This was confirmed by the results of the phase III NOA-8 study in a group of 373 patients with confirmed anaplastic astrocytoma or glioblastoma multiforme, aged >65 years, and with the Karnofsky score of at least 60. A study was conducted to compare single-agent chemotherapy (100 mg/m^2^ of temozolomide, administered on days 1–7 of a cycle, 1 week of treatment, 1 week off treatment) to radiotherapy alone at a dose of 60.0 Gy, administered for 6–7 weeks in 30 fractions of 1.8–2.0 Gy. The overall survival was the primary endpoint. The median overall survival was 8.6 months in the temozolomide group compared to 9.6 months in the radiotherapy group. The methylation of the MGMT promoter was associated with longer overall survival. The most common Grade 3–4 adverse reactions associated with TMZ included hematologic and hepatic toxicity. Infections and thromboembolic events were more frequently observed in the chemotherapy group [85]. Octogenarians may be difficult to qualify for the irradiation of the CNS (central nervous system) metastases from other primary foci.

Nieder et al. presented examples of treatment (surgery, radiosurgery, radiotherapy, systemic therapy) and supportive care in patients >80 years of age with CNS metastases. In the selected group, survival after radiosurgery was longer than after whole-brain radiotherapy. The authors emphasized that, in order to optimize the results, prospective studies focused on octogenarians were necessary [89].

Stadlbauer et al. conducted a retrospective analysis in a group of 94 patients >80 with CNS metastases undergoing whole-brain radiotherapy. The authors proposed an assessment of the survival score based on fitness status, the number of CNS lesions (single or multiple), and the assessment of extracranial disease (present or absent). The median survival was 2.0 months in the whole group. Depending on the score obtained, the patients were assigned to one of 3 groups: poor prognosis (7 points), where all patients died within 2 months and should be qualified for best supportive care (BSC). The intermediate prognosis group obtained 10 points. Those patients also had an unfavorable prognosis with the median survival of 2.0 months and the 3-month OS of 25% and the 6-month survival rate of 13%. BSC was also the best choice in the group. Irradiation at a dose of 5 × 4 Gy should be considered only in selected cases.

In the group of good prognosis (13–17 points), the survival of 6 months was achieved by 50%, and 12 months—by 27% of patients. The authors recommended the following irradiation regimens: RT 5 × 4 Gy; 10 × 3 Gy; 20 × 2 Gy. This simple clinical tool may be useful in clinical practice [90].

Chen et al. presented a retrospective study suggesting that whole-brain RT was associated with increased toxicity compared to SBRT in patients >70 years of age and patients >80 years of age (21% of the study group) with brain metastases. The median OS calculated from the time of the diagnosis of brain metastases was 4.3 months in patients receiving whole-brain RT and 14.4 months in patients receiving SBRT. Patients receiving SBRT were characterized by better tolerability of the treatment. However, such results could be influenced by the selection of patients receiving a given therapy resulting from the stage of the underlying primary disease, the volume of intracranial disease or the general condition. The age >80 was not found to have an impact on the greater toxicity of the treatment [91].

**Table 6:** Glioblastoma & CNS Metastases—Radiotherapy in Older Adults.

Reference	Radiotherapy Approach	Outcomes/Tolerability	Guidelines/Authors’ Comments
Perry et al. [86]	HF-RT 40 Gy/15 fx + adjuvant TMZ vs. RT alone in ≥65 with glioma	Better OS for HF-RT + TMZ vs. RT alone	Combine HF-RT + TMZ for fit older adults
Roa et al. [88]	Very short RT 25 Gy/5 fx (1 wk) vs. 40 Gy/15 fx (3 wks) in elderly/frail newly diagnosed GBM	Non-inferior: median PFS 4.2 mo both arms; median OS 7.9 mo (25 Gy) vs. 6.4 mo (40 Gy); QoL comparable	25 Gy/5 fx is a recommended option for older adults/frail
Nieder et al. [89]	CNS metastases in ≥80: SRS vs. WBRT; other modalities (surgery, systemic, BSC)	SRS associated with longer survival than WBRT in selected cases; need for octogenarian-focused prospective data	Prefer SRS when feasible; individualize by disease burden and performance status
Stadlbauer et al. [90]	WBRT in ≥80 with brain metastases: propose survival score (fitness, lesion number, extracranial disease)	Median OS 2.0 mo; score 7 (poor) → all died ≤2 mo (BSC recommended); score 10 → OS 2 mo, 3-mo OS 25%, 6-mo 13% (also BSC favored); score 13–17 → 6-mo 50%, 12-mo 27%; suggested RT: 5 × 4 Gy/10 × 3 Gy/20 × 2 Gy	Simple clinical score aids WBRT vs. BSC decision and fractionation choice
Chen et al. [91]	WBRT vs. SRS in >70 (including >80 = 21%)	Median OS from brain mets diagnosis: 4.3 mo (WBRT) vs. 14.4 mo (SRS); better tolerability with SRS; age >80 not linked to higher toxicity; selection bias likely	Favor SRS when feasible (limited volume/lesions); avoid routine WBRT in toxicity-sensitive

## 4. Conclusions

The subject of radiotherapy in the group of octogenarians is currently little understood. The data are mainly based on retrospective analyses of small groups of patients.

**Figure 2:**
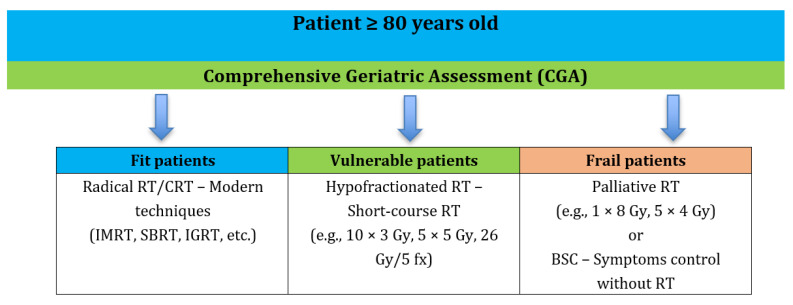
Conceptual overview of radiotherapy decision-making in octogenarian cancer patients.

Treatment recommendations are often extrapolated from other age groups due to the underrepresentation of older adults in clinical trials. Treatment should bring a real benefit to the patient, be well tolerated and organizationally feasible. A significant use of geriatric scales is emphasized to facilitate decision making regarding the choice of the type of therapy for an individual patient. A particular role is attributed to Comprehensive Geriatric Assessment (CGA), which is used to assess various domains regarding the patient, i.e., the clinical, functional, mental, emotional, and social domain. The introduction of CGA as a standard in the assessment of patients allows the individual adjustment of therapy, increasing the effectiveness of treatment and minimizing the risk of side effects. Currently, the CGA scale is rarely used in clinical practice due to its difficulty, complexity and time-consuming nature [13,92]. (Figure 2).

It is necessary to develop new, simple geriatric assessment tools that facilitate the individualization of treatment. We need to develop research on biomarkers and predictors of treatment tolerance in this population. It is also essential to improve communication with the patient and their family regarding the nature of the disease, the treatment process, clear presentation of risks and benefits and finally the making of informed decisions.

Radiotherapy is an effective and safe method of tumor treatment.

SBRT or hypofractionation RT is effective, safe, well tolerated, and leads to a reduction in the total treatment time. These approaches can be used in the treatment of breast, lung, prostate, and rectal cancers, gliomas.

Combined-modality therapy should be reserved for carefully selected patients due to its toxicity, duration, and the adaptive challenges associated with a prolonged therapeutic process.

The benefit of adjuvant therapies with respect to local recurrence or overall survival in this group is uncertain.

The primary limitation of this review stems from its dependence on retrospective data, which are subject to selection bias and may therefore impact the reliability of the findings.

Prospective clinical trials are needed to provide robust scientific evidence on the effectiveness, tolerability, and safety of radiotherapy in late old adults oncological patients, particularly in those with breast, prostate, or rectal cancer. There is a lack of prospective research, including long-term studies assessing distant treatment outcomes. Nevertheless, in this patient population it appears challenging to design and conduct studies that reliably achieve their intended objectives and generate robust data, due in part to factors such as competing mortality and the fact that octogenarians constitute only approx. 10% of all cancer patients populations.

The aging of societies and the increase in the number of patients with neoplastic diseases mean that oncologists more and more commonly face the dilemma of making therapeutic decisions in patients in this age group. It is necessary to constantly expand the knowledge of the oncological treatment, including the late elderly, i.e., individuals older than 80 years of age.

## Data Availability

No new data were created or analyzed in this study.
